# Synergistic killing of lung cancer cells by cisplatin and radiation via autophagy and apoptosis

**DOI:** 10.3892/ol.2014.2049

**Published:** 2014-04-08

**Authors:** MIN LIU, SHUMEI MA, MINGBO LIU, YUFEI HOU, BING LIANG, XU SU, XIAODONG LIU

**Affiliations:** 1 Key Laboratory of Radiobiology (Ministry of Health), School of Public Health, Jilin University, Changchun, Jilin 130021, P.R. China; 2 Department of Radiotherapy, The First Hospital of Jilin University, Changchun, Jilin 130021, P.R. China; 3 Chinese Center for Medical Response to Radiation Emergency, National Institute for Radiological Protection, China Center for Disease Control, Beijing 100088, P.R. China; 4 Department of Radiology and Radiation Oncology, China-Japan Union Hospital, Changchun, Jilin 130021, P.R. China

**Keywords:** cisplatin, radiosensitivity, synergistic killing, lung cancer, autophagy, apoptosis

## Abstract

Cisplatin is a commonly used drug for chemotherapy, however, whether it may be used synergistically with radiotherapy remains unclear. The present study investigated the underlying mechanisms of synergistic killing by radiosensitization and cisplatin, with a focus on the growth inhibition, apoptosis and autophagy of non-small cell human lung cancer cells *in vitro* and in a tumor xenograft *in vivo*. A549 cells were used for the *in vitro* experiments and divided into the following four treatment groups: Sham-irradiated; conventional radiotherapy (CRT) of five doses of 2 Gy every day; hyperfractionated radiotherapy of five doses of 2 Gy (1 Gy twice a day at 4 h intervals) every day; and CRT plus cisplatin. A xenograft tumor-bearing C57BL/6 model was established for the *in vivo* experiments and the above-mentioned treatments were administered. MTT and colony formation assays were used to detect cell viability and western blotting was performed to detect the levels of protein expression. Monodansylcadaverine staining and the immunofluorescence technique were used to analyze the autophagy rate, while flow cytometry and immunohistochemistry were performed to detect the expression levels of the genes associated with apoptosis and autophagy, including microtubule-associated protein 1 light chain 3 (MAPLC3)-II, phosphoinositide 3-kinase (PI3K) III, Beclin1, phosphorylated protein kinase B (p-AKT), damage-regulated autophagy modulator (DRAM), B-cell lymphoma 2 (Bcl-2), Bcl-2-associated X protein, caspase-3 and p21. The MTT assay demonstrated that cisplatin exhibits a dose-dependent cytotoxicity in A549 cells and synergizes with radiation to promote the cell-killing effect of radiation. In the xenograft mouse model of Lewis cells, cisplatin plus ionizing radiation (IR) (five doses of 2 Gy) yielded the most significant tumor suppression. The autophagic vacuoles, the ratio of MAPLC3-II to MAPLC3-I (LC3-II/LC3-I) and the levels of Beclin1 were found to increase in all treatment groups, with the most marked upregulation observed in the CRT plus cisplatin treatment group. In addition, caspase-3 processing was enhanced in the group treated with the combination of cisplatin with radiation, compared with the group treated with radiation alone. Fractionated IR resulted in a significant increase in p21 expression, which was further enhanced when combined with cisplatin. Furthermore, treatment with cisplatin and fractionated IR resulted in a significant elevation of the expression of the autophagy-related genes, PI3KIII, Beclin1 and DRAM1. However, the levels of p-AKT were observed to decline following exposure to fractionated IR in the presence or absence of cisplatin. As for the apoptosis signaling genes, the combination of cisplatin and fractionated IR therapy resulted in a significant decrease in Bcl-2 expression and a marked upregulation of p21 expression. The current study offers strong evidence that the combination of cisplatin with radiation strengthens the killing effect of radiation via pro-apoptotic and pro-autophagic cell death.

## Introduction

Although significant progress has been achieved in the diagnosis and treatment of cancer, lung cancer continues to be the leading cause of mortality worldwide. The overall five-year survival rate of lung cancer is only 15.9% and, at diagnosis, 15% of cases are at the local stage. However, in 22% of cases, the tumor has metastasized to the regional lymph nodes or directly invaded nearby structures and in 56% of cases, distant metastasis has occurred. Therefore, few patients have the opportunity for curative surgery and, thus, the majority of patients can rely only on radiotherapy and chemotherapy (1–3).

During the process of radiotherapy and chemotherapy, multiple changes occur in the growth arrest and death pathways (4–6). Traditionally, irradiation is considered to induce cell death mainly via apoptosis. However, more recent studies have suggested that autophagy is also important in irradiation-induced cell death, which may aid to restore and improve radiosensitivity (7). Autophagy is a highly conserved cellular process whereby intracellular organelles and long-lived proteins are degraded to maintain homeostasis (8). Autophagy has also been revealed to act as a double-edged sword in the initiation, development and metastasis of cancer. On one hand, autophagy exhibits an antitumor role (9); while on the other hand, autophagy, an adaptive response, protects tumor cells against stress. Once autophagy is inhibited, the therapeutic effect is considered to be evidently enhanced (10–20). Cisplatin, a cell cycle non-specific antineoplastic agent, is usually regarded to function as a radiosensitizer during chemoradiotherapy, as it effectively inhibits the repair of sublethal damage resulting from irradiation.

The aim of the present study was to investigate the effect and mechanism of the synergistic killing of lung cancer cells induced by different fractionated radiotherapy treatments, as well as in combination with cisplatin *in vitro* and *in vivo.* The results identified the optimum dose of fractionated radiotherapy and whether the combination of cisplatin may offer a promising approach for clinical application.

## Materials and methods

### Cell culture, antibodies and reagents

The A549 cells and the mouse Lewis lung cancer cell line were cultured in RPMI-1640 and Dulbecco’s modified Eagle’s medium, respectively, supplemented with 10% fetal bovine serum and 1% penicillin-streptomycin at 37°C in a CO_2_ incubator.

Monodansylcadaverine (MDC) was purchased from Sigma-Aldrich (St. Louis, MO, USA). The rabbit polyclonal anti-human antibodies against microtubule-associated protein 1 light chain 3 (MAPLC3), phosphoinositide 3-kinase (PI3K) III, Beclin1, damage-regulated autophagy modulator (DRAM), B-cell lymphoma 2 (Bcl-2), Bcl-2-associated X protein (Bax), caspase-3, p21, p53 and phosphorylated protein kinase B (p-AKT) were purchased from Cell Signaling Technology (Beverly, MA, USA) and the mouse polyclonal antibodies against GAPDH, peroxidase-conjugated anti-mouse IgG and peroxidase-conjugated anti-rabbit IgG were purchased from Santa Cruz Biotechnology, Inc. (Santa Cruz, CA, USA). The Annexin V-FITC Apoptosis Detection Kit I was purchased from BD Biosciences (San Diego, CA, USA).

### Treatment protocol

The A549 cells were divided into the following four groups: Control, no treatment administered; conventional radiotherapy (CRT), five doses of 2 Gy every day; hyperfractionated radiotherapy (HRT), five doses of 2 Gy administered as 1 Gy twice a day at 4 h intervals; and CRT plus cisplatin, five doses of 2 Gy and cisplatin (3 mg/kg intraperitoneally prior to the first irradiation). The radiotherapy was performed using a deep X-ray machine operated at 180 kV/18 mA with 0.25-mm copper filters at a dose rate of 0.41 Gy/min.

### MTT assay

The A549 cells were cultured in 96-well microplates and various concentrations of cisplatin (1, 2.5, 5, 10, 20 and 40 μM) were added to each well for 48 h. A total of 20 μl MTT solution (5 mg/ml) was then added to each well and the plates were incubated for 4 h. The absorbance was measured at 540 nm using a microplate reader.

### Colony formation assay

The cells were trypsinized, counted and plated in 60 mm petri dishes containing standard culture medium. The cells were then treated with various doses of irradiation (0, 2, 4, 6 and 8 Gy) using a 180-KVp X-ray generator at a dose rate of 0.41 Gy/min (200 kV; 18 mA) and cisplatin (5 μM) was added prior to radiation. After two weeks, the cells were stained with crystal violet and the surviving colonies of >50 cells were scored under a dissection microscope (K102-Z; AmScope, Chino, CA, USA). The surviving fraction for each treatment dose was calculated as the plating efficiency of the irradiated samples compared with that of the sham-irradiated samples. For each dose level in the three treatment groups, three independent experiments were performed and the multi-target click model of GraphPad Prism 5.0 (Systat Software, Inc., San Jose, CA, USA) was used to generate cell survival curves.

### MDC staining

The A549 cells were seeded on coverslips overnight and, following the indicated irradiation treatments, 0.5 μM MDC was added to the cells, which were cultured for 1 h. The cells were then washed with phosphate-buffered saline (PBS) and fixed with a solution of 3.3% paraformaldehyde for 30 min. The coverslips were examined using fluorescence microscopy (Olympus XSZ-D2; Olympus Corporation, Tokyo, Japan).

### Flow cytometry analysis

For the analysis of apoptosis, the A549 cells were treated with various doses of ionizing radiation (IR), collected 24 h later and then washed three times with PBS. Next, the cells were stained using the Annexin V-FITC Apoptosis Detection Kit I (BD Biosciences) according to the manufacturer’s instructions. The number of apoptotic cells was determined by flow cytometry (FACSCanto; BD Biosciences) and analyzed using the FCS Express v2.0 software (De Novo Software, Los Angeles, CA, USA).

### Western blot analysis

The total proteins were extracted with radioimmunoprecipitation assay lysis buffer [HEPES (50 mM), NaCl (150 mM), EDTA (1 mM), EGTA (2.5 mM), NaF (10 mM), DTT (1 mM), SV (1 mM), PMSF (1 mM), NP-40 (1%) and SDS (0.1%)] (Jilin University, Changchun, China) and a 2 ml aliquot of the total proteins was mixed with 20 μl protease inhibitor cocktail (Roche Diagnostics GmbH). Next, proteins (40 μg) were separated by SDS-PAGE (Life Technologies, Carlsbad, CA, USA) and transferred to nitrocellulose membranes (GE Healthcare, Arlington Heights, IL, USA) blocked with 5% dry milk or 3% bovine serum albumin in Tris-buffered saline and Tween 20 [10 mmol/l Tris (pH 7.5), 100 mmol/l NaCl and 0.1% Tween 20] both purchased from Shanghai qcbio Science & Technologies Co., Ltd. (Shanghai, China). The membranes were then incubated with primary antibodies and horseradish peroxidase (HRP)-conjugated secondary antibodies. The signals were visualized by chemiluminescence (Western Blotting Luminol Reagent: sc-2048; Santa Cruz Biotechnology, Inc.) and GAPDH was used as a loading control. The intensities of the protein bands were quantified using ImageJ software (US National Institutes of Health, Bethesda, MD, USA) and the ratio of specific band to control was analyzed.

### Tumor-bearing mouse model and treatment protocols

Male C57BL/6 mice, weighing 20±2 g, were purchased from the Animal Center of Chinese Academy of Sciences (Beijing, China). Lewis cells (2×10^5^ cells per mouse in 0.2 ml saline) were injected subcutaneously in the right hind leg of each mouse and the tumors were allowed to grow. When the volume of tumors had reached ~300 mm^3^, the mice were divided randomly into different groups of 10 mice each. Cisplatin (3 mg/kg) was intraperitoneally injected prior to the first dose of radiation. Approval for animal experimentation was obtained from the University Animal Care Committee of Jilin University (Changchun, China).

The four groups of mice were treated as aforementioned; local radiation was administered to the selected areas and the other parts of the mice were protected by lead shielding.

### Measurement of tumor volume and weight

The tumor growth was monitored by measuring the tumor diameters in two dimensions with a caliper each day. The tumor volumes were calculated using the following formula: Tumor volume (mm^3^) = (L × S^2^)/2, where L is the long diameter and S is the short diameter.

The mice were sacrificed by cervical dislocation 24 h following the final treatment and the tumor samples were removed and weighed immediately.

### Immunohistochemical (IHC) staining

The BALB/c mice were sacrificed and the xenografts were removed and fixed with 10% buffered formalin for 16 h, and embedded in paraffin blocks according to a conventional tissue processing procedure (21). The IHC staining was performed on 5-μm-thick sections of the paraffin and tissue microarray blocks. The sections were deparaffinized, rehydrated and then subjected to antigen retrieval, which was performed according to several recommended methods (22). The endogenous peroxidase activity was blocked using 0.3% hydrogen peroxide and the primary antibodies, anti-p-AKT, -MAPLC3-II, -PI3KIII, -Beclin1, -Bcl-2, -Bax, -DRAM and anti-p21, were applied and the samples were further incubated for 90 min at room temperature. The biotinylated homologous secondary antibodies were then applied and the samples were incubated for 30 min at room temperature. Following the reaction with the HRP-labeled streptavidin, diaminobenzene was added for color development followed by counterstaining with hematoxylin. The number of positive cells was counted in five microscopic fields from each tissue slide using ImagePro Plus 5.1 software (Media Cybernetics, Inc., Rockville, MD, USA).

### Terminal deoxynucleotidyl-transferase mediated dUTP nick end labeling (TUNEL) assay

Briefly, the sections were deparaffinized, rehydrated and digested with Proteinase K (Shanghai qcbio Science & Technologies Co., Ltd.) and then labeled with TUNEL reaction mixture (biotin-labeled POD; GenScript, Piscataway, NJ, USA) for 60 min at 37°C. Next, the sections were screened for positive nuclei under a light microscope (Olympus CX21; Olympus Corporation). Data from all fields were pooled to obtain the apoptotic index and are presented as the percentage of TUNEL positive cells in the overall cell population, manually counted in 10 randomly selected fields.

### Statistical analysis

Data are presented as the mean ± standard error and were analyzed by Student’s t-test, one-way analysis of variance or the χ^2^ test using SPSS version 17.10 (SPSS, Inc., Chicago, IL, USA). P<0.05 was considered to indicate a statistically significant difference.

## Results

### Cisplatin increases the cell-killing effects induced by radiation of tumor cells

**Figure 1:**
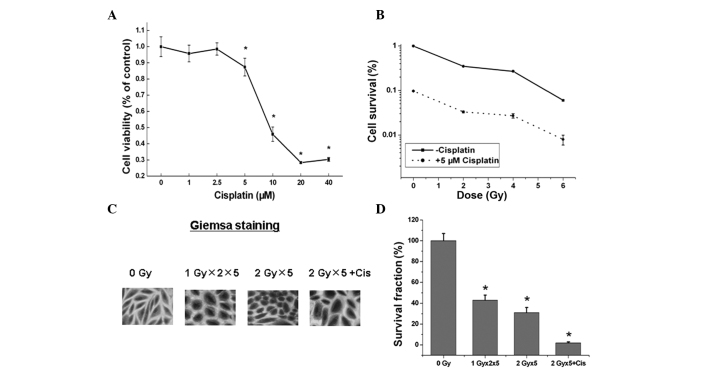
Changes in the survival rate of A549 cells following treatment with cisplatin and radiation. (A) Cell viability was detected by MTT assay following treatment of the A549 cells with various concentrations of cisplatin. (B) The survival fraction was detected by colony formation following the cisplatin (5 μM)treatment of cells with different radiation doses. (C) Morphological changes were observed by Giemsa staining and the (D) survival rate of the A549 cells was detected by colony formation following the different therapeutic regimens.

The A549 cells were seeded onto a 96-well plate when 70–80% confluence was reached and treated with various doses of cisplatin for 48 h. The viability of the cells was then analyzed by MTT assay and cisplatin exhibited dose-dependent cytotoxicity in the A549 cells (P<0.05; Fig. 1A). A colony formation assay was performed to analyze the responses of the A549 cells to radiation with or without 5 μM cisplatin. As shown in Fig. 1B, cisplatin enhanced the radiation-induced cytotoxicity in A549 cells compared with that of the cells treated with IR alone. The colony formation assays and IHC staining identified a similar trend between the cells treated with fractionated IR with and without cisplatin (Fig. 1C and D). These observations indicated that cisplatin potently suppresses A549 tumor cell growth and synergizes with radiation to promote the cell-killing effect of radiation.

### Combination of cisplatin with radiation enhances the cell-killing effects in vivo

**Figure 2:**
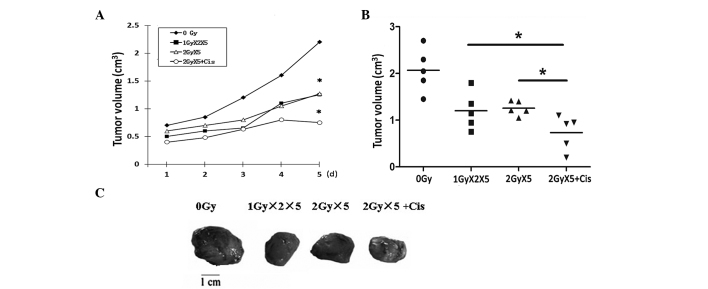
Killing effects of cisplatin and radiation in a xenograft model. (A) Tumor volume curves for the five days after the initiation of treatment. (B) Average tumor volumes in the four treatment groups were measured on the sixth day following the initiation of treatment. In comparison with the control group, the mice in the groups administered with different treatments, in particular the CRT plus cisplatin treatment group, exhibited tumors of smaller size. (C) Xenografts were removed following the different treatments and the tumor sizes were measured.

To verify the effects of cisplatin and radiation on tumor growth *in vivo*, Lewis cells were injected into mice to establish the xenograft model. When the xenografts had grown to the same size, the mice were randomly grouped and treated as previously described. In comparison with the control group, the mice in the groups administered with different treatments, in particular the CRT plus cisplatin treatment group, exhibited tumors of smaller size (Fig. 2). These results provide additional evidence that the combination of cisplatin with IR leads to improved radiotherapy outcome.

### Cisplatin enhances radiation-induced autophagy

**Figure 3:**
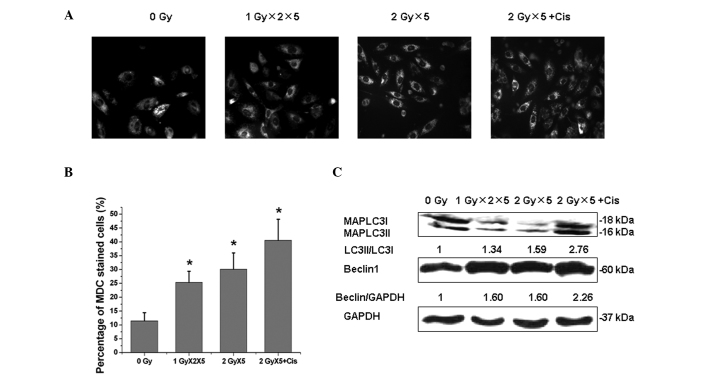
Cisplatin promotes ionizing radiation-induced autophagy. (A) Autophagosomes were detected by MDC staining under fluorescent microscope following the different therapeutic regimens. (B) Statistical analysis of the autophagic rate was performed based on MDC staining and data are presented as the percentage of positive cells (mean ± standard error) of ten fields of vision. ^*^P <0.05, vs. sham-irradiated samples (0 Gy). (C) MAPLC3 and Beclin-1 expression was detected by western blotting, and GAPDH was used as an internal control. MDC, monodansylcadaverine; MAPLC3, microtubule-associated protein 1 light chain 3.

Next, the underlying mechanism of the synergistic effect of cisplatin and radiation was investigated. Autophagy has been shown to exhibit a paradoxical function during cancer radiotherapy under certain circumstances; to confer radioresistance (23,24) or enhance radiation-induced cytotoxicity (25). As shown in Fig. 3, exposure of A549 cells to fractionated IR resulted in a significant elevation of autophagy rates as indicated by MDC staining. In addition, cisplatin was found to promote IR-induced autophagy. During the process of autophagy, the cytoplasmic MAPLC3-I protein (ATG-8 homolog) is converted to a lapidated form, MAPLC3-II, which tightly binds to the autophagosome membrane. In addition, Beclin1 was upregulated in A549 cells following exposure to fractionated IR.

### Cisplatin enhances IR-induced apoptosis

**Figure 4:**
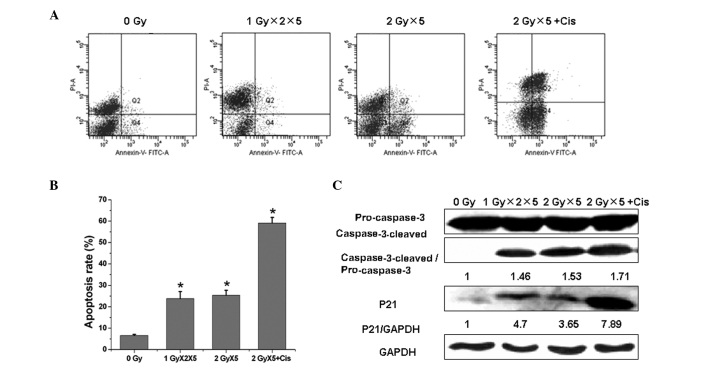
Cisplatin promotes ionizing radiation-induced apoptosis. (A) Apoptosis was determined by flow cytometry following the different treatments and (B) statistical analysis of the apoptotic rate was based on flow cytometry. Data are presented as the percentage of positive cells (mean ± standard deviation) of three experiments.^*^P<0.05, vs. sham-irradiated group (0 Gy). (C) Caspase-3 and p21 expression was detected by western blotting, and GAPDH was used as an internal control.

Apoptosis has been acknowledged to be the predominant killing mechanism following radiation treatment. In the present study, fractionated IR upregulated apoptosis significantly in the A549 cells and cisplatin was found to markedly promote fractionated IR-induced apoptosis (Fig. 4A and B). To elucidate the underlying mechanism of this process, the levels of cleaved caspase-3 in the irradiated cells were evaluated at 48 h in the fractionated IR and fractionated IR plus cisplatin groups (Fig. 4C). The results demonstrated that the caspase-3 protein procession is involved in fractionated IR-induced apoptosis. Furthermore, the production of the active cleaved fragment of caspase-3 following fractionated IR treatment supported the involvement of this protease in apoptosis. Caspase-3 processing was enhanced further following the combination of cisplatin with radiation, when compared with radiation alone.

Fractionated IR was also found to result in a marked increase of p21 expression in the A549 cells, which was further enhanced with the cisplatin and radiation combination treatment. These observations suggested that cisplatin promotes the radiation-induced apoptosis via the activation of caspase-3 protein procession and p21 expression.

### Combined effect of cisplatin and IR on the autophagy regulatory genes

**Figure 5:**
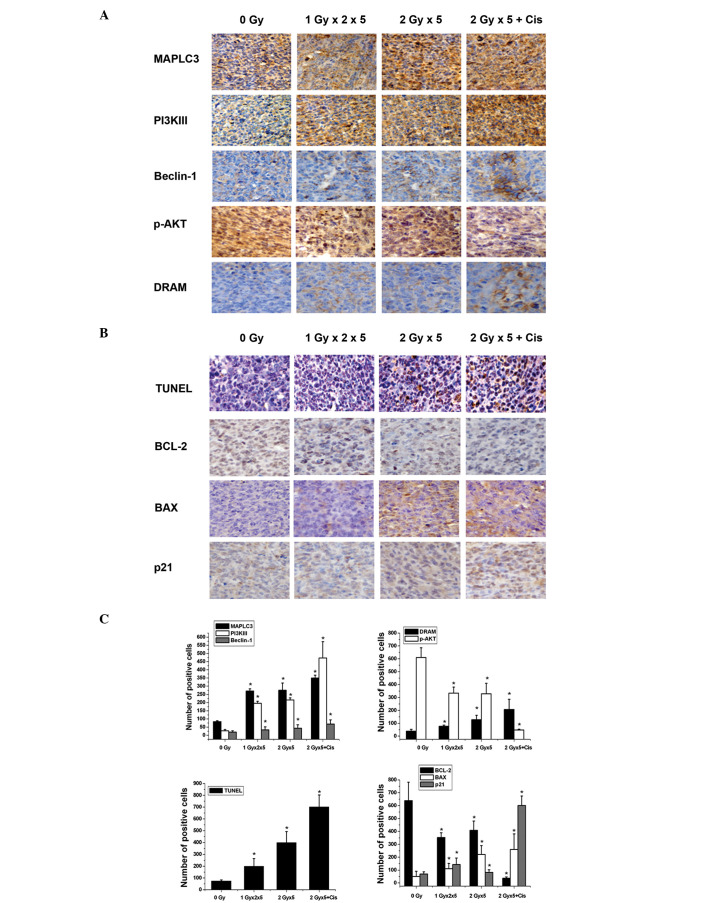
Changes in the expression levels of the apoptosis and autophagy regulatory genes following the different therapeutic regimens. (A) IHC analysis of MAPLC3, PI3KIII, Beclin1, p-AKT and DRAM expression in the xenografts. (B) TUNEL assay was used to detect the apoptotic rate and IHC staining was used to detect BCL-2, BAX and p21 expression in the xenografts. (C) Statistical analysis of the expression of the autophagy and apoptosis related genes was performed based on the IHC staining results. Data are presented as the percentage of positive cells (mean ± standard deviation) of five fields of vision. ^*^P<0.05, vs. sham-irradiated group (0 Gy). IHC, immunohistochemical; MAPLC3, microtubule-associated protein 1 light chain 3; PI3K, phosphoinositide 3-kinase; p-AKT, phosphorylated protein kinase B; DRAM, damage-regulated autophagy modulator; TUNEL, terminal deoxynucleotidyl-transferase mediated dUTP nick end labeling; Bcl-2, B-cell lymphoma 2; Bax, Bcl-2-associated X protein.

Following the confirmation of the association between combination therapy and autophagy, the effect of radiation alone and radiation plus cisplatin on the diverse genes crucial in the autophagy signaling pathways were investigated. As shown in Fig. 5A and C, IHC analysis demonstrated that the combination of cisplatin with fractionated IR results in a significant elevation of PI3KIII and Beclin1 expression levels (P<0.05) when compared with exposure to fractionated IR alone (P<0.05). Class I PI3Ks activate AKT/PKB via phosphorylation, which in turn inhibits autophagy (26,27). In the current study, the level of p-AKT was found to decline following exposure to fractionated IR in the presence or absence of cisplatin. The DRAM1 gene has also been reported to promote the autophagy process (28). In the present study, DRAM1 was markedly upregulated following the exposure of the A549 cells to the combined treatment when compared with radiation alone. In addition, the MAPLC3-II protein (which indicates autophagosome formation) was significantly upregulated following exposure to radiation with or without cisplatin (P<0.05) compared with the Sham-irradiated group. These observations indicated that cisplatin acts synergistically with radiation to trigger autophagic signaling pathways.

### Impact of combination therapy on apoptosis-related genes

Bcl-2 and Bax are crucial components of the apoptotic machinery. As shown in Fig. 5B and C using TUNEL assay and IHC analysis, the combined cisplatin and fractionated IR therapy resulted in a significant decrease of Bcl-2 expression levels (P<0.05) when compared with fractionated IR alone (P<0.05). By contrast, Bax expression was significantly elevated following the different treatments. In addition, p21 expression was markedly upregulated following combined therapy. These results indicated that the combination of cisplatin with radiation affects the apoptosis signaling genes more potently than radiation alone.

## Discussion

Radiotherapy and chemotherapy are effective treatments for lung cancer, which induce tumor cell death via a variety of signaling pathways. Numerous efforts have been made to confirm that apoptosis induces cell death with regularity (10). However, the involvement of autophagy (type II programmed cell death) has not yet been fully clarified in this process.

In contrast to apoptosis, autophagy is paradoxical as it may induce autophagic cell death or provide a survival vehicle for the tumor against nutrition- and energy-deficient environments as a result of chemotherapy, endocrine drugs and irradiation, among others. Cisplatin, a commonly used chemotherapy agent, is generally considered to be a radiosensitizer as it inhibits the repair of sublethal damage from irradiation (29). The aim of the present study was to investigate the mechanism exerted by cisplatin and irradiation on non-small cell lung cancer cells *in vitro* and tumor xenografts *in vivo,* as well as to offer promising evidence for clinical practice.

Firstly, the current study revealed that cisplatin enhances the killing effect of irradiation in A549 cells and a xenograft model. Furthermore, although cisplatin was not found to act as a radiosensitizer, it did exhibit a synergistic effect.

Next, the underlying mechanisms of the synergistic effects of cisplatin and radiation were investigated and the autophagic and apoptotic changes were detected following the different A549 cell treatments. MDC staining and western blotting revealed an increase in autophagosome number, as well as increased MAPLC3 and Beclin1 expression when cisplatin was combined with radiation. Previous studies of autophagy in the human lung cancer cell lines, H460 and A549, provide strong and direct evidence that autophagic vacuoles may be induced by ionizing irradiation and that the autophagic death of tumor cells may be enhanced by an autophagy inducer alone or in combination with ionizing irradiation *in vitro* (13,30,31), which are consistent with the results of the present study. Additionally, data have implied that the inhibition of autophagy potentiates chemosensitivity to cisplatin (32).

Apoptosis is known to be the predominant cell-killing mechanism induced by radiation (33). The results of the present study revealed that cisplatin promotes radiation-induced apoptosis via the activation of the caspase-3 protein procession and p21 expression. Although p21 induces growth arrest and inhibits apoptosis and thereby protects cells in certain systems, studies have also suggested that p21 possesses proapoptotic functions under specific conditions in other systems (34). In thymocytes, the upregulation of p21 leads to hypersensitivity to cell death in response to IR and ultraviolet radiation, but not to dexamethasone in transgenic animals (35).

Following the confirmation in the present study of the association between combination therapy and autophagy/apoptosis, the impact of radiation alone and radiation plus cisplatin on the diverse genes that are considered to have crucial roles in autophagy signaling pathways was investigated in the xenografts. The results showed that the MAPLC3-II/MAPLC3-I conversion ratio and Beclin1 expression levels were increased following the combination of cisplatin with radiation. In addition, it was revealed that the combination of cisplatin with fractionated IR resulted in a significant elevation of the autophagy signaling genes, PI3KIII and Beclin1, as well as declined p-AKT levels and upregulated DRAM1 gene expression. These results demonstrated that cisplatin combined with radiation enhances autophagic cell death. The PI3Ks (classes I and III) are a family of enzymes that are involved in autophagic signaling. Class III PI3Ks have been shown to stimulate autophagy and Beclin1 (also known as Atg6 or BECN1) is an integral protein in the class III PI3K pathway and triggers autophagy. Beclin1 binds to class III PI3Ks and aids in the regulation of the autophagosome formation. Furthermore, studies have shown that following irradiation, the PI3KI/AKT signal pathway has a close association with the repair of DNA damage. The cell stress caused by IR, such as DNA base deletion and glucose molecule damage, may be repaired by DNA polymerase β which may be overexpressed in the PI3KI/AKT signal pathway. Thus, the inhibition of the PI3KI/AKT signaling pathway as a result of the addition of cisplatin improves the cell-killing efficacy of irradiation (36).

In the present study, the combination of cisplatin with fractionated IR was found to effect the apoptosis-related genes, resulting in a significant decrease in Bcl-2 expression and increase in p21 and Bax expression. Bcl-2 is known to negatively regulate the Beclin1-induced autophagy cell death (37) and, therefore, the inhibition of Bcl-2 was considered to potently enhance autophagy. However, Bax expression was significantly elevated following treatment with fractionated IR, but was not elevated with combined therapy. Conversely, p21 expression was markedly upregulated following combined therapy. These results indicated that the combination of cisplatin with radiation affects the apoptosis signaling genes more potently than radiation alone.

In conclusion, the current study offers strong evidence that the combination of cisplatin with radiation strengthens the cell-killing effect of radiation via proapoptotic and proautophagic cell death.
